# [Retracted] Bone morphogenetic protein and activin membrane-bound inhibitor suppress bone cancer progression in MG63 and SAOS cells via regulation of the TGF-β-induced EMT signaling pathway

**DOI:** 10.3892/ol.2026.15941

**Published:** 2026-11-09

**Authors:** Fengsong Liu, Kai Wang, Liang Zhang, Ya-Lin Yang

Oncol Lett 16: 5113-5121, 2018; DOI: 10.3892/ol.2018.9268

Following the publication of this paper, it was drawn to the Editor's attention by a concerned reader that the three data panels showing the results of cell invasion and migration assay experiments in Figs. 2C and D on p. 5116 and 4H on p. 5118 contained multiple overlapping sections, both within the same data panels and comparing across the three figure parts, such that data which were intended to have shown the results from differently performed experiments had been derived from a much smaller number of sources. In addition (and among further problems), a concern was raised that the p16 antibody that the authors reported to have used (cat. no. ab51243) is specific for the protein known as P16 ARC (or ARPC5), not the apoptosis-associated protein p16INK4a, as intended. Furthermore, an independent analysis of the data in this paper undertaken by the Editorial Office revealed that there were also duplicated western blots featured within Fig. 4B, and that data in Fig. 4B, and also histological images in Figs. 1C and 5D, were strikingly similar to data that had already been published, or were already under consideration for publication, in a number of other articles submitted to different journals by different research groups at different research institutes.

Given that the contentious data named above had already been published prior to the receipt of this paper at *Oncology Letters*, and also considering the multiple instances of data having been assembled incorrectly in various of the figures, the Editor has decided that this paper should be retracted from the Journal. The authors were asked for an explanation to account for these concerns, but the Editorial Office did not receive a reply. The Editor apologizes to the readership for any inconvenience caused.

